# Insulin Resistance as a Systemic Metabolic Risk State for Cancer: Mechanisms, Biomarkers, and Prevention

**DOI:** 10.3390/ijms27125495

**Published:** 2026-06-18

**Authors:** Marijana Matek Sarić, Nataša Lisica Šikić, Tamara Sorić, Ana Sarić, Andrija Ivanišin, Ivona Brodić, Mirta Milić

**Affiliations:** 1 Department of Health Studies, University of Zadar, Splitska 1, 23000 Zadar, Croatia; marsaric@unizd.hr (M.M.S.); nlisicasi@unizd.hr (N.L.Š.); 2 Psychiatric Hospital Ugljan, Otočkih dragovoljaca 42, 23275 Ugljan, Croatia; 3 School of Medicine, Catholic University of Croatia, Ilica 242, 10000 Zagreb, Croatia; asaric1@unicath.hr; 4 Independent Researcher, 10000 Zagreb, Croatia; andrija.ivanisin@gmail.com (A.I.); ivona.nutricionist@gmail.com (I.B.); 5 Division of Toxicology, Institute for Medical Research and Occupational Health, Ksaverska cesta 2, 10000 Zagreb, Croatia; mmilic@imi.hr

**Keywords:** insulin resistance, cancer risk, hyperinsulinemia, metabolic biomarkers, metabolic risk state, risk stratification, cancer prevention, GLP-1 receptor agonists, tirzepatide, continuous glucose monitoring

## Abstract

Insulin resistance (IR) is traditionally viewed within the context of type 2 diabetes. However, it increasingly appears to represent a broader systemic metabolic risk state with potential relevance for carcinogenesis. Chronic hyperinsulinemia can activate insulin-like growth factor-1-dependent pathways, including phosphoinositide 3-kinase/protein kinase B/mechanistic target of rapamycin and mitogen-activated protein kinase signaling, promoting cellular proliferation while limiting apoptosis. At the same time, IR is closely linked to oxidative stress, chronic low-grade inflammation, and epigenetic alterations, together shaping a tumor-promoting microenvironment. Epidemiological studies report consistent associations between IR and increased cancer risk, particularly for endometrial, liver, and colorectal cancers. Yet causality remains uncertain and likely varies by tumor type. Notably, metabolic dysfunction may also occur in individuals with normal body mass index (BMI), underscoring the limitations of BMI-based risk assessment. Unlike previous reviews that primarily focused on individual mechanisms or epidemiological associations, this review examines IR as a systemic metabolic risk state by integrating molecular, epidemiological, biomarker-based, and prevention-oriented perspectives. Particular emphasis is placed on strategies for earlier risk identification using integrated biomarker approaches, including fasting glucose, homeostatic model assessment of insulin resistance, triglyceride-to-high-density lipoprotein ratio, high-sensitivity C-reactive protein, and insulin-like growth factor-1. Emerging tools such as continuous glucose monitoring and hepatokine profiling may further refine risk detection. Sustained lifestyle modification—diet, physical activity, sleep, and stress regulation—remains central to prevention. Pharmacological therapies, including glucagon-like peptide-1 receptor agonists and dual incretin agents, offer additional metabolic benefits, although their long-term impact on cancer risk is still unclear. Therefore, IR is best understood not as an isolated risk factor, but as a systemic metabolic risk state that may influence cancer development, with implications for prevention and early risk stratification.

## 1. Introduction

Insulin resistance (IR) has traditionally been viewed within the framework of endocrinology and metabolic disease. Increasing evidence from oncological research suggests that IR represents a broader systemic condition that shapes the biochemical environment of the organism, including processes that may favor carcinogenesis [1,2]. Chronically elevated insulin therefore acts not only as a metabolic regulator, but also as a mitogenic signal. Through activation of the insulin-like growth factor (IGF) pathway, it engages key oncogenic cascades—most notably phosphoinositide 3-kinase/protein kinase B/mechanistic target of rapamycin (PI3K/Akt/mTOR) and mitogen-activated protein kinase (MAPK)—that regulate cellular proliferation, survival, and metabolic reprogramming [2,3,4].

Under physiological conditions, insulin primarily signals through its own receptor (InsR) to maintain metabolic homeostasis, including glucose uptake, glycogen synthesis, and lipid storage. In contrast, insulin-like growth factor 1 (IGF-1) binds predominantly to the IGF-1 receptor and hybrid InsR/IGF-1 receptor complexes, mediating stronger mitogenic and anti-apoptotic effects [5]. In states of chronic hyperinsulinemia, persistently elevated insulin can cross-activate IGF-1 receptors, effectively shifting signaling from a predominantly metabolic role toward a growth-promoting one. This shift provides a biologically plausible mechanism through which prolonged IR may contribute to cellular survival and proliferation under conditions where normal regulatory constraints would otherwise apply. It also has therapeutic implications, as selective IGF-1 receptor inhibition may offer a more targeted approach than broad receptor blockade, which risks disrupting metabolic homeostasis [6,7].

Importantly, IR rarely occurs in isolation. It is typically accompanied by chronic low-grade inflammation, oxidative stress, and dysregulated lipid metabolism, together creating a tumor-promoting microenvironment characterized by DNA damage, enhanced angiogenesis, and progressive tumor development [8]. Epigenetic reprogramming adds a further layer of complexity. Persistent hyperinsulinemia has been associated with histone hyperacetylation and altered DNA methylation patterns, potentially priming gene expression programs that favor cell survival and proliferation even before somatic mutations arise [9,10].

Epidemiological findings are broadly consistent with these mechanisms. IR has been associated with increased incidence of several cancers, including breast, pancreatic, liver, colorectal, and lung cancer, although the strength and consistency of these associations vary across populations and study designs [7,11,12]. These uncertainties have led to increasing interest in biomarker-based approaches for earlier identification of IR and related cancer risk. From a health systems perspective, earlier detection may also have economic implications, as progression to advanced metabolic disease and cancer is associated with substantially higher long-term healthcare costs [13,14].

A clinically important consideration is that IR, visceral adiposity, and low-grade inflammation may also be present in individuals with normal body mass index (BMI). This phenotype, often referred to as normal-weight metabolic obesity (NWO), challenges the adequacy of BMI-based screening and highlights the need for more comprehensive metabolic assessment [15,16].

Large observational cohorts such as the UK Biobank, the European Prospective Investigation into Cancer and Nutrition (EPIC), and the Women’s Health Initiative (WHI) provide important insights into the relationships between lifestyle, metabolic dysfunction, and disease risk [17,18,19,20]. Across these cohorts, high-calorie diets, physical inactivity, and obesity are consistently associated with worsening IR and increased cancer risk, whereas plant-based dietary patterns and favorable body composition appear protective [21,22]. These data are subject to confounding, self-report bias, and selection effects, and therefore require cautious interpretation.

These considerations have direct implications for prevention and clinical management. Pharmacological intervention alone—despite clear metabolic benefits—cannot substitute for sustained lifestyle modification. High-calorie diets, physical inactivity, poor sleep, and chronic stress act as upstream drivers of metabolic dysfunction and may contribute to tumor development. Pharmacotherapy can mitigate downstream effects, but does not address these underlying determinants. This is particularly relevant for incretin-based therapies, whose benefits often diminish after discontinuation in the absence of behavioral change, and whose long-term oncological implications remain uncertain [23,24].

The concept of adiponcosis further contextualizes the relationship between IR and cancer by describing how excess adipose tissue contributes to the development of obesity-related malignancies [25,26,27]. Although the underlying biological pathways vary by tumor type and fat distribution, key mechanisms—including IR, hyperinsulinemia, increased IGF signaling, and chronic inflammation—are consistently implicated. Epidemiological data indicate that obesity is associated with increased risk of at least 13 cancer types, and that the burden of obesity-related cancers has risen substantially in recent decades [25,28].

Recent reviews have summarized the role of IR in cancer development and its contribution to tumorigenesis [7]. However, the present review adopts a broader perspective by considering IR as a systemic metabolic risk state that integrates epidemiological, molecular, biomarker-based, and preventive dimensions within a single framework. Particular attention is given to metabolic dysfunction in individuals with normal BMI, integrated biomarker approaches for early risk identification, clonal hematopoiesis of indeterminate potential (CHIP)-associated inflammation, gut microbiome alterations, epigenetic mechanisms, and the evolving role of incretin-based therapies. This review also critically evaluates the strengths and limitations of the current evidence and discusses the translational implications for early risk stratification, prevention, and clinical practice. By combining mechanistic insights with emerging approaches to risk assessment and prevention, this review aims to provide a contemporary framework for understanding the potential role of IR in cancer development. The term “systemic metabolic risk state” is used throughout this review to denote a metabolic context that is epidemiologically associated with increased cancer risk and mechanistically plausible as a contributor to carcinogenesis, rather than an established causal determinant.

## 2. Literature Search

This narrative review was conducted using established approaches for qualitative evidence synthesis. A comprehensive literature search was performed in PubMed/MEDLINE, Web of Science, and Scopus, covering publications from January 2000 to March 2026. Earlier landmark studies were included where relevant to provide historical context.

The search strategy combined Medical Subject Headings (MeSH) and free-text terms. Core concepts included insulin resistance (“insulin resistance”, “insulin sensitivity”, “hyperinsulinemia”), cancer risk (“cancer”, “neoplasms”, “carcinogenesis”, “tumor development”), metabolic dysfunction (“metabolic syndrome”, “obesity”, “metabolic dysfunction”), biomarkers (“HOMA-IR”, “fasting insulin”, “IGF-1”, “triglyceride-glucose index”, “TG/HDL ratio”, “high-sensitivity C-reactive protein”), lifestyle interventions (“diet”, “physical activity”, “exercise”, “sleep”, “lifestyle modification”), and pharmacological approaches (“GLP-1 receptor agonists”, “semaglutide”, “liraglutide”, “tirzepatide”, “dual incretin agonists”). Boolean operators (AND, OR) were used to combine search terms. Reference lists of selected articles were also screened to identify additional relevant studies.

Eligible studies included peer-reviewed original research articles, systematic reviews, meta-analyses, randomized controlled trials, prospective cohort studies, and Mendelian randomization (MR) studies published in English. Case reports, conference abstracts without full-text availability, and studies lacking sufficient methodological information were excluded.

Priority was given to systematic reviews, meta-analyses, large prospective cohort studies, MR studies, and randomized controlled trials when available. Mechanistic and experimental studies were included when necessary to explain biological pathways relevant to IR and carcinogenesis.

Although no formal risk-of-bias assessment tool was applied because of the narrative nature of the review, study quality was considered during evidence synthesis. Evidence was interpreted according to study design, methodological rigor, sample size, and consistency with the broader literature. Consequently, findings from smaller observational studies and preclinical investigations were interpreted more cautiously than those from higher-level evidence sources.

As this is a narrative review without a formal protocol, evidence was synthesized thematically rather than quantitatively. Where possible, we interpreted the evidence by considering the consistency of reported associations and approximate effect sizes across studies. When multiple independent cohorts reported comparable hazard ratios (HRs) for a given cancer type, we summarized the direction and general range of these estimates. Findings that were based on limited evidence or showed inconsistency across studies were interpreted cautiously and discussed in the context of their methodological limitations.

When conflicting findings were encountered, greater emphasis was placed on evidence from meta-analyses, large prospective cohorts, and MR studies because of their stronger ability to address confounding and reverse causality. Discrepancies between studies were interpreted in the context of differences in study populations, exposure definitions, biomarker selection, follow-up duration, and statistical adjustment strategies.

**Figure 1:**
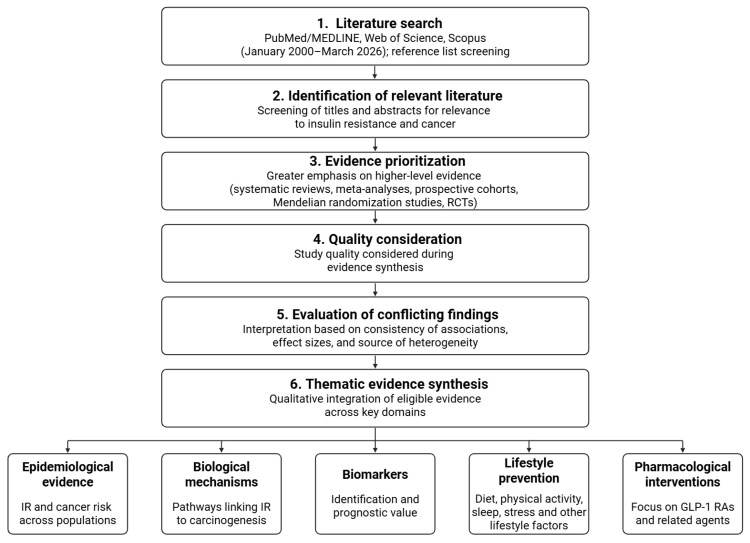
Flow diagram of the literature search and study selection process used in this narrative review. Created in BioRender. Milić, M. (2026) https://BioRender.com/nmba72i. Abbreviations: IR, insulin resistance; RCTs, randomized controlled trials; GLP-1 RAs, glucagon-like peptide-1 receptor agonists.

The literature search and study selection process are summarized in Figure 1.

## 3. Epidemiological Evidence

### 3.1. Association Between Insulin Resistance and Cancer Risk

Epidemiological evidence generally points in the same direction, although distinguishing cause from consequence remains methodologically challenging. The most pronounced associations are observed for gastrointestinal, pancreatic, and hepatic cancers, which is biologically plausible given insulin’s central role in hepatic and gastrointestinal metabolism.

#### 3.1.1. Gastrointestinal Cancers

Evidence from prospective cohort studies consistently suggests a link between metabolic dysfunction and gastric cancer risk. In a large Korean prospective cohort (*n* = 108,397; Health Examinees-Gem study, 2004–2017), metabolic syndrome was associated with a 26% higher risk of gastric cancer compared with metabolically healthy individuals, with a clear dose–response relationship across the number of metabolic syndrome components [HR 1.26; 95% confidence interval (CI) 1.07–1.47] [29]. Similar patterns were observed in a nationwide retrospective cohort in Korea (*n* = 318,336), where increasing quartiles of the metabolic score for IR (METS-IR) were associated with progressively higher gastric cancer incidence after adjustment for major confounders [30].

Comparable findings have been reported in European populations. In a Norwegian prospective cohort (*n* = 192,903; mean follow-up 10.6 years), metabolic syndrome was associated with increased risk of gastric adenocarcinoma (HR 1.44; 95% CI 1.14–1.82), whereas no association was observed for esophageal carcinoma. Among individual components, elevated waist circumference, hypertension, and non-fasting glucose showed independent associations with gastric adenocarcinoma risk in women [31].

These results point to a consistent directional association across populations, but their interpretation requires caution. The Korean cohorts are derived from relatively homogeneous populations with a high prevalence of *Helicobacter pylori* infection, a major independent risk factor for gastric cancer, which may limit generalizability [32].

Emerging approaches combining polygenic risk scores (PRS) with MR analyses offer a way to disentangle inherited susceptibility from acquired metabolic dysfunction, providing a more refined framework for interpreting these associations [33].

#### 3.1.2. Pancreatic Cancer

The relationship between IR and pancreatic cancer is more difficult to interpret. Tumor-induced disruption of islet function complicates the temporal sequence, as metabolic abnormalities observed close to diagnosis may reflect disease effects rather than pre-existing risk. It is estimated that nearly 80% of patients exhibit impaired glucose tolerance at diagnosis [34].

This highlights a key methodological challenge: distinguishing pre-existing IR from tumor-driven dysglycemia. Addressing this requires long pre-diagnostic follow-up or study designs less susceptible to reverse causality, such as MR analyses based on genetic instruments measured before disease onset. Existing MR studies provide limited but informative evidence. A genome-wide analysis using PanScan and PanC4 data suggested that genetically elevated fasting insulin may be associated with increased pancreatic cancer risk, although confidence intervals were wide and findings were not consistent across sensitivity analyses [35].

An umbrella review of MR studies reached a similar conclusion, identifying suggestive associations for fasting insulin but not for fasting glucose or hemoglobin A1c (HbA1c), underscoring the importance of distinguishing IR from hyperglycemia in epidemiological analyses [11].

Taken together, the available evidence suggests a possible link, but one that remains vulnerable to reverse causality and measurement limitations.

#### 3.1.3. Colorectal and Hepatocellular Cancers

A similar pattern of uncertainty is observed for colorectal and hepatocellular cancers. Increased incidence has been reported in populations characterized by high caloric intake and sedentary behavior [36,37,38,39], supporting a role for metabolic dysfunction. However, genetically informed analyses provide a more nuanced picture.

MR studies in postmenopausal women have not demonstrated a direct association between genetically instrumented IR and overall colorectal cancer risk, although potential effect modification by obesity and dietary patterns has been suggested [33]. These findings reflect a broader pattern seen across studies: IR is rarely an isolated exposure, and its apparent effects may depend on the wider metabolic and behavioral context.

#### 3.1.4. Methodological Considerations and Limitations

Epidemiological evidence supports an association between IR and increased risk of gastrointestinal and pancreatic cancers, although causality remains uncertain. Observational studies cannot fully disentangle IR from coexisting factors, and commonly used measures such as the homeostatic model assessment of insulin resistance (HOMA-IR) capture only part of the metabolic phenotype and are subject to short-term variability [40,41,42,43].

Variability in how IR is defined and measured further complicates comparison across studies and may contribute to inconsistent findings. In this context, MR approaches offer an important methodological advantage, but their interpretation depends on several assumptions, including the validity of the genetic instruments and the absence of substantial pleiotropy. Because genetic variants associated with IR often influence related metabolic traits such as adiposity, lipid metabolism, and inflammation, complete separation of the independent effects of IR remains challenging. In addition, limited statistical power may affect analyses of less common cancer subtypes [44].

MR findings are not uniform across cancer types. For pancreatic cancer, genetically elevated fasting insulin has shown suggestive associations with increased risk, although findings were not consistently supported across sensitivity analyses and confidence intervals remained wide [11,35]. In contrast, MR evidence for colorectal cancer remains limited and does not consistently support a direct causal relationship between IR and cancer risk [33]. Similarly, MR studies of lung cancer have generally provided weaker support for a direct causal role of metabolic factors than suggested by observational studies, highlighting the potential contribution of residual confounding, particularly smoking-related factors [45,46]. Therefore, these findings suggest that the causal contribution of IR is likely heterogeneous across cancer types and that observational associations should not automatically be interpreted as evidence of direct causality.

Across the studies reviewed, the overall direction of association between proxy measures of IR and cancer risk is generally consistent, although effect sizes vary by cancer type. For gastric cancer, risk increases of approximately 26–44% have been reported in cohorts with metabolic syndrome (HR 1.26–1.44), with evidence of dose–response relationships [29,31]. Evidence for pancreatic cancer remains suggestive but inconsistent [11,35], while associations for colorectal and hepatocellular cancers are biologically plausible but not consistently supported in MR analyses [33].

The strongest and most consistent epidemiological associations are observed for gastrointestinal and endometrial cancers, whereas findings for lung and breast cancer are weaker or less consistent in genetically informed analyses. In addition, IR and hyperinsulinemia have been associated with increased endometrial cancer risk in prospective biomarker studies and meta-analyses [47,48,49]. Differences between pooled observational estimates and MR findings likely reflect residual confounding, particularly by smoking in the case of lung cancer. For example, pooled observational analyses have reported an approximately twofold increase in lung cancer risk among individuals with IR or related metabolic abnormalities (RR 2.35, 95% CI 1.55–3.58) [12].

MR studies, which are less susceptible to such confounding, suggest that these associations should be interpreted cautiously and not automatically assumed to reflect direct causal relationships. Future MR studies stratified by smoking status and tumor subtype are needed to clarify these associations [44]. The available MR evidence suggests that the contribution of IR to cancer risk is heterogeneous across tumor types, reinforcing the need to distinguish observational associations from causal relationships and to interpret epidemiological findings within their broader metabolic context. No single IR marker [HOMA-IR, fasting insulin, triglyceride–glucose (TyG) index] has demonstrated consistently superior predictive performance across cancer types.

#### 3.1.5. Cancer-Specific Heterogeneity in the Relationship Between IR and Cancer Risk

Although the overall direction of association between insulin resistance and cancer risk is broadly consistent across epidemiological studies, the strength of support and the underlying biological mechanisms differ substantially among tumor types. Endometrial and hepatocellular cancers show the most consistent epidemiological associations, whereas findings for pancreatic, colorectal, and breast cancers are more heterogeneous. Mendelian randomization studies further suggest that the contribution of insulin resistance to cancer risk varies across malignancies, with stronger support for some tumor types than others. These differences have important implications for risk stratification, biomarker development, and prevention strategies. A comparative overview of the major cancer types discussed in this review is provided in Table 1.

### 3.2. Evidence from Large Population Cohort Studies

Large prospective cohort studies are central to epidemiological research on diet, metabolic health, and chronic disease risk. Among the most influential are EPIC, the UK Biobank, and the WHI. Each offers a slightly different perspective, and together they provide a useful—though imperfect—picture of how metabolic dysfunction relates to long-term disease outcomes.

#### 3.2.1. EPIC Cohort

EPIC was designed to examine associations between diet, lifestyle, and cancer risk across diverse European populations. It includes approximately 521,000 participants aged 35–70 years recruited from 23 centers in 10 countries, making it one of the largest resources for prospective cancer research [19,61]. One of its main strengths is precisely this diversity, which allows comparison of dietary patterns and lifestyle behaviors across populations [61,62]. Participants have been followed for decades, enabling assessment of cancer incidence and cause-specific mortality [19,62].

That same diversity introduces complications. Dietary assessment methods differ across countries, and most data are self-reported. Both factors increase measurement error and make it harder to compare results directly [63].

#### 3.2.2. UK Biobank

The UK Biobank offers a complementary type of dataset. It includes over 500,000 participants aged 40–69 years, with detailed phenotypic data, biological samples, and linkage to electronic health records [17]. This combination allows researchers to explore genetic, environmental, and lifestyle determinants of disease in a way that smaller cohorts cannot.

In practice, the UK Biobank has been particularly useful for studying dietary determinants of IR and type 2 diabetes (T2D), where relatively modest associations can still be detected because of the large sample size. At the same time, interpretation is not straightforward. Participants tend to be healthier, more educated, and of higher socioeconomic status than the general population—a classic “healthy volunteer” effect that limits generalizability [17,64]. In addition, more than 94% of participants are of white British ancestry, which further restricts applicability to more diverse populations. As in EPIC, reliance on self-reported dietary data introduces recall and reporting bias.

#### 3.2.3. WHI Cohort

The WHI provides a different perspective, focusing specifically on postmenopausal women. The cohort includes 93,676 participants aged 50–79 years recruited across 40 clinical centers in the United States between 1993 and 1998 [65]. Selection criteria excluded individuals with conditions predictive of short-term mortality, which improved internal validity but reduced representativeness [20,65].

As is often the case in large cohorts, the effective sample size varies depending on the analysis. Exclusions (for example, prior cancer diagnoses) and attrition reduced the number of participants in specific studies—for instance, to 79,990 in breast cancer analyses [65]. Follow-up averages around 10.3 years, with some participants followed for up to 18 years in hormone therapy trials, allowing assessment of long-term outcomes [66]. WHI also includes longitudinal data on diet and metabolic outcomes such as IR and T2D.

Like the other cohorts, WHI is observational. This means that confounding remains an issue, and differences between observational findings and randomized trial results—particularly in the context of hormone therapy—highlight the limits of causal interpretation [67,68].

#### 3.2.4. Methodological Considerations and Limitations

Despite their differences, these cohorts share several limitations. One of the most consistent is reliance on self-reported dietary data, which introduces both measurement error and reporting bias.

Another issue is how metabolic exposure is captured. Many analyses rely on a single measurement—often baseline HOMA-IR—which may not reflect long-term metabolic status. Over long follow-up periods, this can weaken observed associations if changes over time are not accounted for.

A more fundamental limitation is that IR itself is rarely measured directly. Instead, it is inferred from diet, anthropometric measures, or downstream outcomes such as T2D or metabolic syndrome. Direct measurements, such as fasting insulin or HOMA-IR, are usually available only in sub-cohorts. As a result, many associations attributed to IR likely reflect broader metabolic dysfunction rather than a clearly defined intermediate state.

Considered together, EPIC, the UK Biobank, and WHI provide valuable longitudinal evidence linking metabolic dysfunction to chronic disease. Their shared limitations—self-reported data, indirect measurement of IR, single time-point assessments, and limited population diversity—make causal interpretation difficult.

For the IR–cancer association specifically, analyses from EPIC report RRs in the range of approximately 1.1–1.6 for several cancer types when obesity or hyperinsulinemia proxies are used, with stronger associations for endometrial (RR ~ 2.0), liver, and colorectal cancers [69,70]. These effect sizes are broadly consistent with findings from other large cohorts. Because IR is not directly measured, these estimates may underestimate risk in individuals with metabolically unfavorable phenotypes.

### 3.3. Metabolic Risk in Individuals with Normal BMI

BMI remains one of the most widely used population-level measures because it is simple to calculate and easy to interpret. Its limitations are equally well recognized. BMI does not distinguish fat mass from lean mass, nor does it reflect adipose tissue distribution. As a result, individuals with a normal BMI may still carry substantial cardiometabolic risk despite apparently normal weight.

Metabolic risk is therefore not uniform within the normal BMI range. Luo et al. [15] reported a graded relationship between BMI trajectory and T2D risk, with gradual BMI increase associated with an adjusted risk ratio of 1.34 and rapid increase with a risk ratio of 2.06. Increasing attention has also been directed toward the concept of NWO, defined by normal BMI in combination with excess body fat and metabolic dysfunction. NWO has been associated with increased risk of postmenopausal breast cancer (HR 1.19), as well as endometrial and colorectal cancers [57,58].

This disconnect between BMI and metabolic health is also reflected in population-based studies. Suliga et al. [71] reported metabolic syndrome in 17.27% of normal-weight individuals in Poland, while metabolic dysfunction-associated steatotic liver disease (MASLD) has been observed in approximately 19% of lean individuals [16]. Reported prevalence estimates vary considerably depending on diagnostic definitions and study design, making direct comparisons difficult.

The limitations of BMI-based assessment become even clearer at the molecular level. CHIP has emerged as an area of growing interest that may provide insight into the relationships among metabolic dysfunction, chronic inflammation, and cancer risk [72,73]. However, its role in mediating the association between IR and cancer remains incompletely understood and requires further validation. CHIP is characterized by recurrent somatic mutations in epigenetic regulators, most commonly DNA methyltransferase 3A (*DNMT3A*), tet methylcytosine dioxygenase 2 (*TET2*), and additional sex combs-like 1 (*ASXL1*) [72]. These mutations alter transcriptional regulation in hematopoietic stem and progenitor cells and promote expansion of pro-inflammatory clones [73]. Some of the strongest mechanistic evidence involves *TET2* loss-of-function mutations, which enhance inflammatory signaling and increase secretion of interleukin-6 (IL-6) and interleukin-1β (IL-1β), both central mediators of chronic low-grade inflammation [74]. *DNMT3A* mutations similarly influence cytokine expression through altered DNA methylation patterns [73]. Together, these mutation-driven changes provide a biologically plausible framework through which CHIP could contribute to cardiometabolic and oncological risk. Current evidence is largely derived from mechanistic and observational studies, and further longitudinal and experimental investigations are needed to establish the clinical relevance and causal significance of these associations [72,73,75]. This further illustrates why reliance on BMI alone may be insufficient when evaluating long-term metabolic risk. At present, CHIP should be considered an emerging and hypothesis-generating concept rather than an established mechanistic link between IR and cancer.

These findings highlight a clinically important disconnect between BMI and metabolic dysfunction. They support moving beyond BMI-based classification toward a broader metabolic phenotype approach incorporating anthropometric measures, body composition, and biochemical markers. Among simple screening tools, the waist-to-height ratio (WHtR; threshold ≥ 0.5) has shown relatively consistent predictive performance across populations because it better reflects central adiposity [76].

Important limitations nevertheless remain. Clinical guidance on integrated non-BMI screening strategies is still limited, and no major diabetes or oncology guideline currently recommends systematic WHtR-based cancer risk stratification. This reflects the broader lack of prospective studies evaluating combined screening approaches integrating anthropometric and metabolic markers.

These observations suggest that BMI-based stratification may underestimate the burden of cancer risk associated with IR. They also support the use of more comprehensive approaches to metabolic risk assessment in future epidemiological and clinical studies. From here, an important question follows naturally: which biological mechanisms connect IR and cancer development?

## 4. Biological Mechanisms

### 4.1. Hyperinsulinemia and Activation of the IGF-1/PI3K-Akt-mTOR Pathway

The PI3K/Akt/mTOR pathway is one of the major regulators of cellular metabolism, growth, and proliferation and is therefore central to discussions of hyperinsulinemia-driven IR. Chronic insulin exposure can activate this pathway through both insulin and IGF-1 receptors, leading to phosphorylation of downstream targets such as proline-rich Akt substrate of 40 kDa (PRAS40) and ribosomal protein S6 kinase beta-1 (p70S6K1), with downstream effects on cellular proliferation and survival [59,77]. Pathway activation reflects more than insulin signaling alone. Growth factors, oncogenic mutations, and microenvironmental stress signals all converge on PI3K/Akt/mTOR activity, making its regulation highly context-dependent.

Insulin and IGF-1 signal through closely related pathways, although their physiological roles differ. Insulin is primarily involved in metabolic regulation, whereas IGF-1 exerts stronger mitogenic and anti-apoptotic effects in most tissues. This distinction becomes relevant in cancer, where circulating IGF-1 concentrations are often elevated and many tumors overexpress IGF-1 receptors or hybrid insulin/IGF-1 receptors [59]. As a result, malignant cells may become increasingly sensitive to systemic metabolic signals.

Persistent activation of the PI3K/Akt/mTOR pathway has been particularly well described in breast and endometrial cancers [59,60,78]. This pathway is also among the most frequently somatically altered signaling networks across solid tumors. Mutations involving *PIK3CA*, *AKT1*, or the tumor suppressor gene *PTEN* are common. *PIK3CA* mutations, for example, occur in approximately 30–40% of breast cancers, *PTEN* loss/alteration is reported in about 45–50% of endometrial carcinomas, and *AKT1* mutations are reported in roughly 5–10% of several solid tumor types [50,51,79]. These observations suggest that any effects of IR-related hyperinsulinemia are likely to interact with pre-existing genetic alterations and tumor-specific signaling programs rather than act independently. Although these observations support biological plausibility, direct evidence that hyperinsulinemia-driven pathway activation independently initiates human carcinogenesis remains limited.

Another important issue is timing. Reversal of IR through lifestyle intervention may improve metabolic status without fully reversing the epigenetic landscape established during years of chronic hyperinsulinemia [9,10]. Chronic mTOR activation also promotes phosphorylation of p70S6K1, which contributes to degradation of insulin receptor substrate-1 (IRS-1) and further worsens IR through negative feedback signaling [80,81]. This may partly explain why clinical mTOR inhibitors (rapalogs) can aggravate hyperglycemia despite targeting a pathway closely linked to metabolic dysfunction.

**Figure 2:**
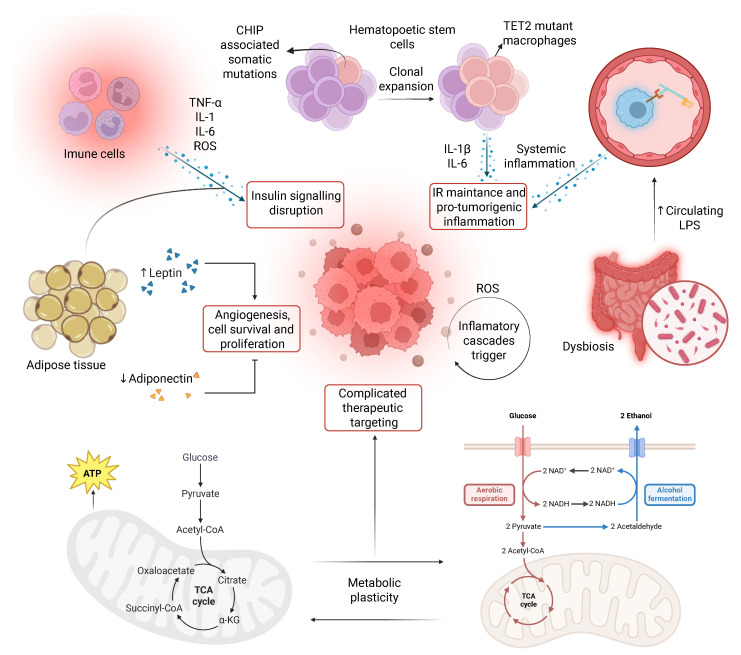
Tumor-promoting microenvironment arising from insulin resistance. Note: Schematic overview of the mechanisms through which insulin resistance contributes to the establishment of a tumor-promoting microenvironment. Key features of insulin resistance, including adipokine imbalance (increased leptin and decreased adiponectin), chronic low-grade inflammation, CHIP, and gut dysbiosis, are associated with increased production of pro-inflammatory cytokines and ROS. CHIP-associated somatic mutations and dysbiosis-derived LPS may further amplify systemic and pro-inflammatory signaling. While growing evidence suggests that CHIP could contribute to chronic inflammation and metabolic dysfunction, its role in insulin resistance-associated carcinogenesis remains emerging and requires further validation. Adipokine imbalance contributes to angiogenesis, cellular survival, and proliferation, whereas metabolic plasticity enables adaptation to changing metabolic demands, supporting tumor growth and complicating therapeutic targeting. Created in BioRender. Milić, M. (2026) https://BioRender.com/8ldytns. Abbreviations: TNF, tumor necrosis factor; IL, interleukin; ROS, reactive oxygen species; CHIP, clonal haematopoiesis of indeterminate potential; *TET2*, tet methylcytosine dioxygenase 2; LPS, lipopolysaccharide; TCA, tricarboxylic acid cycle; ATP, adenosine triphosphate; NAD^+^, nicotinamide adenine dinucleotide; NADH, reduced nicotinamide adenine dinucleotide.

Figure 2 illustrates the major components of the tumor-promoting microenvironment arising from IR and their potential interactions.

### 4.2. The Tumor-Promoting Microenvironment

The tumor-promoting microenvironment does not arise from a single mechanism. Instead, it reflects interactions among chronic inflammation, oxidative stress, mitochondrial dysfunction, adipokine imbalance, and metabolic reprogramming toward glycolysis. These processes are closely interconnected, although their relative importance likely varies across tissues and tumor types.

Chronic inflammation is one of the main links between IR and carcinogenesis. Inflammatory signaling may be triggered by infections, environmental exposures, or metabolic disturbances such as IR itself, leading to release of pro-inflammatory cytokines and reactive oxygen species (ROS), tissue injury, and increased cellular proliferation [8,82]. Cytokines including tumor necrosis factor-α (TNF-α), interleukin-1 (IL-1), and IL-6 are produced by adipose tissue and immune cells and can impair insulin signaling while further promoting IR [83,84]. Tumors can also induce systemic inflammatory and metabolic changes, highlighting the bidirectional nature of these interactions.

Oxidative stress represents another important component of this environment. Excess ROS production combined with impaired antioxidant defense mechanisms can disrupt insulin signaling, impair glucose uptake, and activate inflammatory pathways [85,86]. This creates a self-reinforcing cycle in which oxidative stress and inflammation amplify one another. At lower concentrations ROS also function as signaling molecules involved in normal cellular homeostasis, meaning that their biological effects remain context-dependent.

Mitochondrial dysfunction provides another point of overlap between metabolic disease and cancer. Impaired oxidative phosphorylation can increase reliance on glycolysis even under aerobic conditions, a phenomenon classically described as the Warburg effect. In many tumors mitochondria remain functionally active, and the shift toward glycolysis appears to reflect metabolic adaptation rather than complete mitochondrial failure [82,87]. A somewhat similar metabolic shift occurs in IR and T2D, although in this setting it is generally compensatory rather than proliferative. Tumor cells also retain substantial metabolic flexibility, switching between glycolytic and oxidative states depending on substrate availability, hypoxia, and oncogenic signaling. This metabolic flexibility may partly explain why therapeutic responses vary across tumor types.

Adipose tissue contributes to this environment through secretion of adipokines. Leptin, typically elevated in obesity and IR, activates oncogenic pathways including Janus kinase/signal transducer and activator of transcription 3 (JAK/STAT3) and PI3K/Akt signaling, promotes angiogenesis, and has been linked to progression of breast and colorectal cancers [4,56]. Adiponectin shows an opposing pattern. Although produced by adipocytes, circulating adiponectin levels decrease in obesity, and its biological effects include activation of AMP-activated protein kinase (AMPK), inhibition of mTOR signaling, and anti-proliferative activity [4,88].

The leptin-to-adiponectin ratio has consequently been proposed as a marker of adipose tissue dysfunction with potential oncological relevance, although its clinical utility remains uncertain because standardized reference ranges are lacking [4]. These adipokine alterations are best viewed as part of a broader tumor-promoting microenvironment rather than isolated mediators.

Interest has also shifted toward the gut–tumor microenvironment axis. Dysbiosis associated with IR—characterized by reduced microbial diversity, altered *Firmicutes*-to-*Bacteroidetes* ratios, and impaired intestinal barrier integrity—may increase circulating lipopolysaccharide (LPS) levels and activate toll-like receptor 4 (TLR4)-dependent inflammatory pathways [53,54,55]. The resulting low-grade endotoxemia contributes to both IR and systemic inflammation. In contrast, microbiota enriched in butyrate-producing species may improve metabolic regulation and reduce colorectal cancer risk through short-chain fatty acid (SCFA)-mediated signaling.

As discussed in Section 3.3, CHIP has emerged as a potential contributor to chronic inflammation and metabolic dysfunction. Mutations involving *TET2*, *DNMT3A*, and *ASXL1* have been associated with enhanced inflammatory signaling, including increased production of cytokines such as IL-6 and IL-1β, which may contribute to impaired insulin signaling [72,73,74]. Emerging evidence also suggests that IR and related metabolic disturbances may favor clonal expansion [89,90], raising the possibility of a bidirectional relationship. However, the precise role of CHIP in linking IR with cancer development remains incompletely understood [91,92]. Current evidence is derived primarily from mechanistic and observational studies, and further longitudinal and experimental investigations are needed to determine whether CHIP acts as an independent mediator or represents part of a broader inflammatory axis [73,93].

These mechanisms are highly interconnected and operate across multiple levels of biological organization. The distinction between InsR and IGF-1 receptor signaling, for example, has therapeutic implications, and selective IGF-1 receptor inhibition is being explored as a strategy to reduce mitogenic signaling while preserving metabolic regulation [6]. The coexistence of inflammation, oxidative stress, adipokine imbalance, dysbiosis, mitochondrial dysfunction, and epigenetic alterations suggests that no single pathway is likely to be solely responsible for IR-associated carcinogenesis. This redundancy may partly explain the limited efficacy of interventions targeting single pathways.

At present, no controlled human studies directly quantify the relative contribution of these mechanisms to cancer risk in individuals with IR, and much of the available evidence therefore remains preclinical or inferential. Prospective mechanistic studies embedded within metabolic intervention trials are needed to clarify these relationships. Consequently, most mechanistic evidence should be interpreted as supporting biological plausibility rather than establishing direct causality.

**Figure 3:**
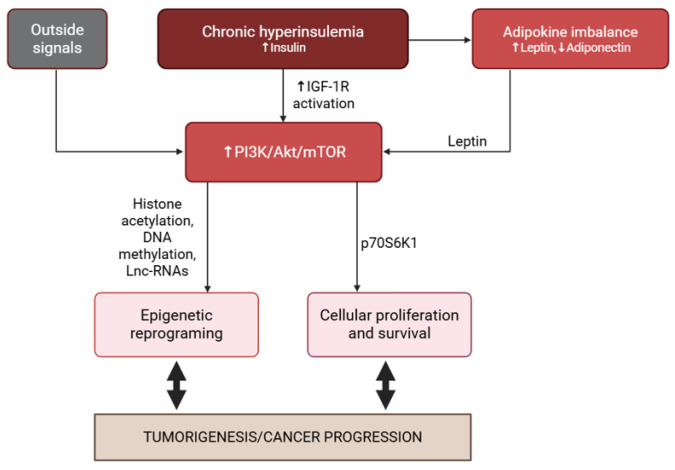
Mechanism linking chronic hyperinsulinemia to tumorigenesis through activation of the IGF-1R/PI3K/Akt/mTOR signaling pathway. Note: While metabolic abnormalities may be partially reversible through lifestyle interventions, epigenetic alterations can persist even after metabolic normalization. Created in BioRender. Milić, M. (2026) https://BioRender.com/2asbg8m. Abbreviations: IGF-1R, insulin-like growth factor-1 receptor; PI3K/Akt/mTOR, phosphatidylinositol 3-kinase/protein kinase B/mechanistic target of rapamycin; Lnc-RNA; long non-coding RNA; p70S6K1, p70 ribosomal S6 kinase 1.

Figure 3 illustrates how chronic hyperinsulinemia may contribute to tumorigenesis through activation of the IGF-1R/PI3K/Akt/mTOR signaling pathway and its downstream effects on cellular proliferation, survival, and metabolic reprogramming.

The biological pathways discussed in this section provide plausible mechanisms through which IR and chronic hyperinsulinemia may contribute to carcinogenesis. However, most evidence derives from experimental, preclinical, or observational studies. Therefore, mechanistic findings should be interpreted alongside epidemiological and genetically informed studies when evaluating the oncological significance of IR.

Because these processes interact at multiple levels, identifying individuals at highest risk will likely require biomarker approaches capable of capturing multiple dimensions of metabolic dysfunction.

## 5. Early Biomarkers and Risk Identification

### 5.1. Key Metabolic Biomarkers

Before discussing individual biomarkers, it is important to distinguish between three partially overlapping categories of evidence: (1) biomarkers validated for diabetes risk assessment and metabolic monitoring; (2) biomarkers that primarily provide mechanistic insight into pathways linking insulin resistance and carcinogenesis; and (3) emerging biomarkers whose clinical applicability remains investigational [94]. The biomarkers discussed in this section span all three categories, and their current level of evidence should be interpreted accordingly. Unless explicitly stated otherwise, references to clinical applicability in this section relate to metabolic risk assessment rather than cancer risk prediction specifically. Early identification of individuals at risk of T2D, metabolic syndrome, and related long-term complications depends on biomarkers that capture different aspects of metabolic function. In clinical and research settings, five markers are used most often: fasting glucose, HOMA-IR, the triglyceride-to-high-density lipoprotein ratio (TG/HDL), high-sensitivity C-reactive protein (hs-CRP), and IGF-1 [95]. Together, they reflect related but non-identical dimensions of metabolic risk: glycemic regulation, hepatic insulin sensitivity, atherogenic dyslipidemia, low-grade inflammation, and growth factor-mediated signaling [96,97]. This grouping is based on clinical practicality and biological plausibility rather than formal validation against all possible biomarker combinations [94]. Table 2 summarizes established and emerging biomarkers, including their biological roles, clinical applicability, main limitations, and current level of evidence.

No single biomarker captures the full complexity of metabolic dysfunction. Fasting glucose reflects current glycemic status, but it is influenced by acute stress, intercurrent illness, and deviations from fasting conditions [43,98]. In an intervention analysis, Parast et al. [42] reported that fasting glucose accounted for approximately 80.1% of the observed effect on diabetes incidence. HOMA-IR showed a slightly lower contribution of 77.7%, but provides more direct information on hepatic insulin resistance. Commonly used HOMA-IR cut-off values are >2.5 in European populations and 2.0–2.5 in Asian populations, although thresholds vary by age, sex, population, and assay methodology. A further limitation is that reported HOMA-IR thresholds vary according to population characteristics, including age, sex, ethnicity, and study design, which limits direct comparison across studies. These cut-off values should therefore be understood as population-specific thresholds rather than universal criteria, and their applicability to cancer risk stratification has not been prospectively validated [3,99,100].

HbA1c is useful for monitoring longer-term glycemic exposure because it reflects average glucose levels over approximately three months. For early risk detection, however, it is less sensitive. The American Diabetes Association notes that HbA1c may fail to identify a substantial proportion of individuals with impaired fasting glucose, supporting the use of complementary biomarkers in early assessment [43]. The TG/HDL ratio provides a practical marker of atherogenic dyslipidemia and correlates with IR [101]. Its predictive performance varies according to ethnicity, sex, and underlying metabolic phenotype, limiting the applicability of a single universal cut-off value across diverse populations [99,100]. hs-CRP adds information on low-grade systemic inflammation, although it is not specific and may be elevated in a range of inflammatory or acute conditions [102,103]. IGF-1 completes the core panel because it links metabolic dysfunction with growth factor signaling and oncogenic pathway activation [5,104,105]. Recent mechanistic studies further support a role for IGF-1-related signaling in obesity-associated tumor–metabolic crosstalk, particularly in breast cancer, where interactions among adipocytes, stromal cells, and tumor cells may contribute to disease progression and treatment resistance [106,107]. Circulating IGF-1 shows substantial biological variability related to age, sex, nutritional status, hepatic function, and sampling conditions [108,109]. Therefore, IGF-1 is best regarded as a mechanistically informative research biomarker rather than a validated clinical screening tool for cancer risk prediction.

Several emerging biomarkers may further refine metabolic risk assessment. Fibroblast growth factor 21 (FGF-21), a hepatokine induced by metabolic stress, is elevated in insulin-resistant states and has been associated with progression to T2D independently of conventional markers [110,111]. Fetuin-A, a liver-derived glycoprotein, can impair insulin receptor signaling and is elevated in metabolic syndrome, with reported associations involving visceral adiposity and cancer risk [112,113]. Retinol-binding protein 4 (RBP4), secreted by adipocytes and hepatocytes, contributes to IR in skeletal muscle and liver and represents another candidate biomarker [114,115]. These markers are not currently recommended in clinical guidelines, but they may prove useful in research settings, particularly in individuals with normal BMI and subclinical metabolic dysfunction.

CGM has added a more dynamic dimension to metabolic risk assessment in non-diabetic populations. Unlike fasting glucose or HbA1c, CGM captures postprandial excursions, glycemic variability, and time-in-range metrics. This is relevant because individuals classified as normoglycemic by standard criteria may still exhibit substantial glycemic variability. For example, glucose levels in apparently healthy individuals have been reported to reach prediabetic ranges for up to 15% of monitored time [116]. A systematic review also found increased glycemic variability in individuals with prediabetes, together with inverse correlations between variability and beta-cell function [117].

These findings suggest that CGM-derived metrics may improve identification of early metabolic dysregulation and support more refined metabolic phenotyping [117,118]. The clinical significance of CGM-detected variability in otherwise healthy individuals remains uncertain. Longitudinal studies are still needed to determine whether these patterns predict future metabolic disease or cancer-related outcomes. Thus, CGM-derived metrics in non-diabetic or otherwise metabolically healthy individuals remain exploratory, and reference thresholds for cancer risk prediction have not been established.

Multi-marker approaches are attractive because they capture several biological dimensions simultaneously, but they also introduce practical challenges. Cost, laboratory availability, assay standardization, and interpretation all influence feasibility. A combined approach incorporating glycemic markers, measures of IR, and indicators of beta-cell function is likely to provide the most informative assessment [110,119]. Even then, such panels do not capture genetic susceptibility, socioeconomic factors, or cumulative lifetime exposure to metabolic risk. Integration with polygenic risk scores and longitudinal clinical data may eventually support more precise prevention strategies.

Economic considerations are also relevant. Expanded biomarker panels and CGM-based approaches may increase short-term healthcare costs, although earlier detection and prevention of advanced metabolic disease could reduce long-term costs [120,121].

The biomarkers discussed in this section differ substantially in their level of clinical validation and applicability. Fasting glucose and HOMA-IR are most relevant for diabetes risk assessment and metabolic monitoring, although HOMA-IR remains limited by assay and population variability. The TG/HDL ratio and hs-CRP provide complementary information on dyslipidemia and systemic inflammation, but neither has been prospectively validated for cancer risk prediction. IGF-1, FGF-21, fetuin-A, and CGM-derived metrics are best regarded as mechanistic or exploratory markers whose translational potential requires validation in adequately powered and diverse prospective cohorts. To date, no biomarker panel combining these markers has been validated as a cancer risk screening tool in metabolically at-risk, non-diabetic populations.

**Table 2:** Established and emerging biomarkers of insulin resistance: clinical applicability, limitations, and current level of evidence.

Marker	What It Measures	Clinical Advantage	Limitation	Current Level of Evidence
Validated panel (5 markers)				
Fasting glucose [42]	Current glycemic regulation	Explains ~80% of intervention effect on diabetes incidence	Sensitive to acute factors; does not directly reflect IR	Validated for diabetes risk assessment and metabolic monitoring; not cancer-specific
HOMA-IR [42]	Hepatic insulin sensitivity	Derived from fasting glucose and insulin; reflects underlying IR	Single measurements do not capture long-term exposure; variability across populations and assays	Useful for IR quantification and diabetes risk research; not standardized for cancer risk prediction
TG/HDL ratio [119]	Atherogenic dyslipidemia	Practical surrogate marker of IR; widely available and cost-effective	Indirect measure; influenced by ethnicity and metabolic heterogeneity	Practical metabolic and cardiovascular risk marker; performance varies by population
hs-CRP [22]	Low-grade systemic inflammation	Established marker for cardiovascular risk; associated with metabolic dysfunction	Low specificity; elevated in infection, trauma, and inflammatory conditions	Validated inflammatory risk marker; non-specific for cancer risk prediction
IGF-1 [5]	Growth factor-mediated and mitogenic signaling	Mechanistic link between metabolic dysfunction and oncogenic pathways	Age- and sex-dependent variability; requires standardized sampling conditions	Mechanistic research biomarker; not validated for routine cancer risk screening
Emerging markers (research-level)				
FGF-21 [110]	Hepatokine reflecting metabolic stress and lipid metabolism	Associated with IR and predicts progression to T2D independently of conventional markers	Not standardized; cut-off values vary; not included in clinical guidelines	Exploratory/research-level biomarker
Fetuin-A [112]	Hepatic protein modulating insulin receptor signaling	Associated with visceral adiposity and MASLD; linked to metabolic dysfunction	Influenced by inflammatory states; lack of standardized thresholds	Exploratory/research-level biomarker
CGM—glycemic variability [117]	Dynamic glucose regulation; postprandial excursions	Detects glycemic variability and subclinical dysregulation not captured by static markers	High cost; lack of established thresholds for non-diabetic populations	Exploratory in non-diabetic populations; thresholds for cancer risk prediction not established

Abbreviations: IR, insulin resistance; HOMA-IR, Homeostatic Model Assessment of Insulin Resistance; TG/HDL ratio, triglyceride-to-high-density lipoprotein ratio; hs-CRP, high-sensitivity C-reactive protein; IGF-1, insulin-like growth factor 1; FGF-21, fibroblast growth factor 21; T2D, type 2 diabetes; MASLD, metabolic dysfunction-associated steatotic liver disease; CGM, continuous glucose monitoring.

### 5.2. Potential and Limitations of Early Biomarker Detection

Early biomarker-based detection of metabolic dysfunction and cancer is increasingly viewed as an important component of prevention and risk stratification. Detecting disease before progression to stages with limited therapeutic options is generally associated with improved prognosis and survival [122,123]. In colorectal cancer, for example, biomarkers such as adenomatous polyposis coli (*APC*), tumor protein p53 (*TP53*), and Kirsten rat sarcoma viral oncogene homolog (*KRAS*) mutations can support earlier intervention and have been linked to improved clinical outcomes [124,125,126].

The expanding use of omics technologies—including genomics, proteomics, and metabolomics—has further broadened the range of detectable biomarkers. Within metabolomics, plasma profiles characterized by elevated branched-chain amino acids (BCAAs; leucine, isoleucine, valine), acylcarnitines, and aromatic amino acids have been associated with incident T2D up to a decade before diagnosis [110]. These findings suggest that metabolomic approaches may identify early metabolic risk states not captured by conventional markers. At the same time, reproducibility remains a major limitation. Biomarker panels validated in one cohort often fail to replicate in independent populations because of inter-laboratory variability, pre-analytical differences, and population heterogeneity [127]. This remains one of the main barriers to clinical translation.

The potential benefits of early detection therefore need to be interpreted cautiously. Biomarker-based screening also carries a risk of overdiagnosis and identification of indolent abnormalities that may never become clinically relevant [128,129]. HOMA-IR illustrates this problem well. Measurements obtained in non-fasting states or during acute illness may overestimate IR, potentially leading to unnecessary interventions, patient anxiety, and increased healthcare utilization [130]. Biomarker performance also varies across populations, disease subtypes, and analytical methods, further complicating interpretation [129].

Practical and ethical considerations create additional challenges. Many biomarker-based approaches require specialized laboratory infrastructure and technical expertise that remain unavailable in routine clinical settings, particularly in resource-limited environments. Ethical concerns—including informed consent, data privacy, and the psychological consequences of early risk disclosure—further complicate implementation [131,132]. Communicating probabilistic metabolic risk to asymptomatic individuals is not straightforward and may contribute to unnecessary medicalization or anxiety, especially in populations with limited health literacy [131]. Importantly, elevated biomarker levels generally reflect modifiable risk rather than deterministic outcomes.

Institutional frameworks governing genetic and metabolomic data also remain incompletely developed in many healthcare systems, raising unresolved questions regarding data ownership, secondary use, and potential insurance discrimination [133,134]. Global applicability is further complicated by disparities in healthcare capacity. In low- and middle-income countries, where the burden of IR-related disease continues to rise, access to fasting insulin assays, CGM devices, and omics platforms is often limited by cost, infrastructure, and workforce constraints. In these settings, implementation strategies emphasizing accessible measures such as WHtR, fasting glucose, and the TG/HDL ratio may prove more feasible and equitable [110].

An additional issue is that early biomarker detection is often discussed as though it serves a single clinical purpose, when in reality several distinct goals are involved. These include: (1) detection of preclinical IR before development of T2D; (2) identification of cancer risk associated with metabolic dysfunction; and (3) monitoring response to lifestyle or pharmacological interventions. The evidence supporting these applications is uneven. It is strongest for early detection of metabolic dysfunction, moderate for treatment monitoring, and still limited for prediction of cancer risk itself. Although several biomarkers show mechanistic and epidemiological links to carcinogenesis, no biomarker panel has yet undergone prospective validation as a cancer risk screening tool in metabolically at-risk, non-diabetic populations.

These limitations reinforce the broader point that biomarker-based risk identification should complement, rather than replace, preventive strategies focused on lifestyle and long-term metabolic health. Given the current uncertainties surrounding early detection, prevention remains central to reducing the long-term burden of IR and its downstream consequences.

## 6. Nutrition and Lifestyle in the Prevention of Insulin Resistance

### 6.1. Dietary Strategies for Improving Insulin Sensitivity

#### 6.1.1. Dietary Patterns and Macronutrient Quality

Diet is one of the most accessible approaches for reducing IR, although its effects are closely intertwined with other lifestyle factors. Physical activity, sleep quality, and stress regulation all influence glucose homeostasis, insulin signaling, inflammatory pathways, and body composition. Because of this, metabolic improvement rarely depends on diet alone. Evidence consistently shows that multi-domain lifestyle interventions are more effective than isolated or short-term strategies, while single dietary modifications often produce only modest and transient effects [135,136].

Among dietary approaches, the Mediterranean and DASH patterns remain the most consistently studied and supported for improving insulin sensitivity. Both emphasize high intake of fruits, vegetables, whole grains, legumes, and unsaturated fats, while limiting processed foods, saturated fats, and excess sodium. Their metabolic benefits are likely multifactorial and involve dietary fiber, favorable fatty acid composition, and a broad range of micronutrients and bioactive compounds that influence insulin signaling and systemic inflammation [135,136,137].

Part of these effects may be mediated through the gut microbiota. Diets rich in plant-based foods have been associated with greater abundance of butyrate-producing bacteria such as *Faecalibacterium prausnitzii* and *Roseburia* species [138]. Increased production of short-chain fatty acids (SCFAs), particularly butyrate, has been linked to improved insulin sensitivity through activation of FFAR2/FFAR3 receptors, enhanced GLP-1 secretion, and reduced intestinal permeability, thereby lowering endotoxemia-associated inflammation [139]. This may partly explain why whole dietary patterns often outperform isolated macronutrient-based interventions.

Randomized controlled trials lasting 6–24 months report reductions in HOMA-IR of approximately 0.5–1.5 units with Mediterranean diet interventions, corresponding to relative reductions of roughly 15–30% from baseline [140,141]. Long-term adherence remains a major limitation and varies substantially across populations. A meta-analysis by Gu et al. [142] found higher adherence among younger adults (<45 years) and women. More limited dietary changes, such as increasing fruit and vegetable intake without broader dietary restructuring, generally produce less consistent improvements in IR markers [143].

Carbohydrate quality also appears to influence metabolic outcomes. Diets emphasizing low glycemic index foods promote slower glucose absorption, attenuated insulin responses, and greater metabolic stability. Legume-rich, low-glycemic-index dietary patterns have been associated with reduced fasting leptin concentrations in insulin-resistant individuals [144,145]. Reducing intake of ultra-processed foods (UPFs) is similarly important. UPFs are typically energy-dense, rich in refined carbohydrates and unhealthy fats, and low in dietary fiber, contributing to oxidative stress, chronic low-grade inflammation, and excessive caloric intake [146,147]. High consumption among children and adolescents further emphasizes the importance of prevention early in life.

Protein source may also contribute to long-term metabolic risk. Large cohort analyses suggest that replacing 5% of total energy intake from animal protein with plant-derived protein is associated with lower T2D risk, partly through inflammatory and microbiome-related pathways [22]. Notably, these associations persist after adjustment for BMI, again suggesting that metabolic risk cannot be explained solely by body weight.

#### 6.1.2. Emerging Dietary Approaches and Bioactive Compounds

Emerging dietary approaches such as intermittent fasting (IF) and time-restricted eating (TRE) have received increasing attention in recent years. Protocols including 16:8 TRE and the 5:2 model may provide metabolic benefits that extend beyond simple caloric restriction. Proposed mechanisms include improved circadian alignment, reduced postprandial insulin exposure, and enhanced hepatic insulin sensitivity [142,148]. A meta-analysis by Gu et al. [142] reported comparable—and in some studies greater—improvements in fasting insulin and HOMA-IR relative to continuous caloric restriction.

The evidence base remains limited by relatively short follow-up periods, with most intervention studies lasting less than 12 months. Adherence may also vary substantially, and these approaches are not appropriate for all populations, particularly individuals with eating disorders, frailty, pregnancy, or certain chronic conditions.

Interest has also grown in specific bioactive compounds, particularly polyphenols found in berries, tea, cocoa, and olive oil. These compounds may exert modest metabolic effects through antioxidant and anti-inflammatory activity as well as modulation of glucose-related signaling pathways [149]. Their isolated clinical impact appears limited. Benefits are generally more consistent when such compounds are consumed within broader dietary patterns rather than as individual supplements or targeted interventions.

One important limitation of current dietary intervention research is that cancer incidence is rarely included as a primary endpoint. As a result, links between improvements in IR and reduced cancer risk remain largely inferential and are based mainly on epidemiological associations rather than direct interventional evidence. Clarifying these relationships will require large-scale, long-term studies incorporating both metabolic and oncological outcomes [150].

#### 6.1.3. Lifestyle Modification and Pharmacological Support

In individuals with more advanced or long-standing metabolic dysfunction, lifestyle measures alone may not produce sufficient clinical improvement. Under these circumstances, pharmacological therapy may become an important adjunct to dietary modification and physical activity. Studies consistently show that combining nutritional intervention with regular exercise produces greater improvements in insulin sensitivity than diet alone [151,152].

When lifestyle-based approaches are insufficient, pharmacological treatment is generally introduced as supportive therapy rather than as a substitute for behavioral change. Metformin remains the most widely used first-line agent for T2D and IR. Its primary effects involve reduction in hepatic glucose production and improvement of glycemic regulation through mechanisms that are now well established [153,154].

Glucagon-like peptide-1 receptor agonists (GLP-1RAs) represent a different therapeutic approach. In addition to improving glycemic control, they promote weight reduction through combined endocrine and central nervous system effects that influence satiety and energy intake [155]. Clinical benefits can be substantial, particularly in individuals with obesity-related metabolic dysfunction. These effects are closely tied to treatment continuation. Discontinuation is frequently followed by partial or marked metabolic rebound, including weight regain and deterioration of previously improved cardiometabolic parameters [24,156].

These observations reinforce a broader point seen throughout metabolic intervention research: long-term outcomes depend less on short-term therapeutic intensity than on sustained behavioral and metabolic change. Pharmacological therapy may improve the metabolic environment and facilitate lifestyle adherence, but it does not fully address the upstream drivers of IR when used in isolation.

### 6.2. Physical Activity and Metabolic Regulation

Regular physical activity remains one of the most effective non-pharmacological strategies for improving insulin sensitivity. Exercise influences metabolic regulation through several complementary mechanisms. It increases expression and translocation of glucose transporter type 4 (GLUT-4) in skeletal muscle, facilitating insulin-independent glucose uptake; improves mitochondrial oxidative capacity; reduces visceral adipose tissue while preserving or increasing muscle mass; and promotes greater reliance on lipid oxidation, thereby limiting accumulation of lipid intermediates that interfere with insulin signaling [157].

Aerobic and resistance exercise each provide distinct metabolic benefits, and programs combining both modalities generally produce the most consistent improvements in IR and overall metabolic health [158,159,160]. The relationship between exercise and insulin sensitivity also appears to be dose-dependent. Moderate-intensity activity is associated with measurable metabolic improvement, although thresholds likely exist below which effects become clinically negligible. At the opposite extreme, excessive exercise and overtraining may chronically elevate cortisol levels and impair metabolic regulation [160].

One particularly accessible approach is low-intensity aerobic exercise, often approximated in practice by “Zone 2” training, corresponding to roughly 60–70% of maximum heart rate. This type of exercise has been associated with improvements in mitochondrial function, fatty acid oxidation, and metabolic flexibility in skeletal muscle [161,162,163]. Because mechanical strain is relatively low, it is often better tolerated in sedentary or metabolically compromised individuals than higher-intensity programs.

Sedentary behavior itself represents an additional and partly independent metabolic risk factor. Even individuals who meet recommended exercise targets may remain at increased risk of IR when prolonged sitting occupies much of the day—the so-called “active couch potato” phenomenon [164]. This suggests that structured exercise alone may not fully compensate for sustained sedentary behavior. Clinical counseling should therefore address both planned physical activity and reduction in total sitting time.

As with dietary interventions, most exercise studies have focused primarily on metabolic endpoints rather than cancer incidence itself. Consequently, links between exercise-induced improvements in IR and reduced cancer risk remain indirect. Future studies incorporating multi-domain lifestyle interventions and long-term oncological outcomes may provide a more realistic picture of how physical activity influences the broader metabolic environment associated with carcinogenesis.

Management of IR therefore requires an individualized and staged approach in which sustained lifestyle modification remains central, while pharmacological support is incorporated when clinically necessary.

### 6.3. Sleep, Circadian Rhythms, and Stress as Metabolic Determinants

Chronic psychological stress and poor sleep quality are closely interconnected. Stress can disrupt sleep, while insufficient or fragmented sleep increases physiological stress reactivity and further impairs metabolic regulation [165]. Sleep duration below seven hours per night has consistently been associated with increased risk of IR and T2D, partly through dysregulation of the hypothalamic–pituitary–adrenal (HPA) axis [166]. Sustained activation of this axis leads to chronically elevated cortisol levels, which impair insulin signaling, increase hepatic gluconeogenesis, and promote accumulation of visceral adipose tissue [167,168].

Behavioral mechanisms also contribute to this relationship. Stress-related eating behaviors, particularly increased intake of highly palatable energy-dense foods, represent an additional pathway linking psychological distress with metabolic dysfunction [169].

Circadian regulation adds another layer of metabolic control. Both short and excessively long sleep duration have been associated with increased risk of obesity and T2D, suggesting that sleep quality and circadian alignment are relevant beyond total sleep time alone [170]. Melatonin plays a central role in synchronization of circadian rhythms, and disruption of its secretion has been linked to reduced insulin sensitivity [171].

Meal timing appears to be important as well. Consuming a large proportion of daily caloric intake later in the evening is associated with impaired glucose tolerance and lower insulin sensitivity, whereas earlier food intake tends to produce more favorable glycemic responses [172,173,174]. This likely reflects circadian variation in peripheral metabolic responses, with tissues during the biological evening less prepared to manage postprandial glucose loads. Early time-restricted eating, aligned predominantly with morning and midday hours, is based on this principle and has shown improvements in IR markers independent of weight loss [142].

Interventions targeting sleep and stress may therefore contribute to metabolic improvement, although their effects are generally modest when used in isolation. Cognitive behavioral therapy for insomnia (CBT-I), for example, has been associated with small but measurable improvements in glycemic control [175]. While reductions in HbA1c observed in intervention studies are relatively limited, they may still become clinically relevant when combined with broader lifestyle interventions.

These observations reinforce the idea that metabolic regulation extends beyond diet and exercise alone. Circadian and behavioral determinants are increasingly recognized as important components of metabolic health, although they remain underappreciated in routine clinical practice.

### 6.4. Life-Course Vulnerability and Metabolic Risk

Metabolic responses to stress and sleep disturbances vary across the life course, and certain periods appear particularly sensitive to development of IR and long-term metabolic dysfunction. Pregnancy and the postmenopausal transition are especially important because both involve major hormonal changes that influence insulin sensitivity and cardiometabolic risk [176].

Pregnancy represents a physiological state of substantial metabolic adaptation. A degree of IR normally develops to support fetal growth and nutrient availability. In predisposed individuals this adaptive process may progress to gestational diabetes mellitus (GDM), which is associated with increased long-term risk of T2D and cardiovascular disease in both mother and offspring [177]. Sleep disturbances during pregnancy may further aggravate IR through HPA-axis dysregulation and altered cortisol signaling [178].

The postmenopausal period represents another phase of increased metabolic vulnerability. Declining estrogen levels favor visceral adipose tissue accumulation and reduced insulin sensitivity, contributing to higher cardiometabolic risk and increased susceptibility to several obesity-related cancers [68,166].

Children and adolescents have also emerged as an increasingly important at-risk population. High intake of ultra-processed foods, combined with sedentary behavior and insufficient sleep, contributes to early development of IR and metabolic syndrome [179]. Puberty itself is characterized by a transient physiologic insulin-resistant state, making this period particularly sensitive to metabolic programming and environmental influences [178,180].

### 6.5. Maintaining Healthy Body Composition and the Role of Targeted Supplementation

Body weight management remains an important component of IR prevention and treatment, although fat distribution appears more relevant than body weight alone. Visceral adipose tissue is more strongly associated with IR and cardiometabolic risk than subcutaneous fat. As discussed earlier, individuals with normal BMI may still exhibit metabolically unfavorable fat distribution, reinforcing the limitations of BMI-based assessment and the importance of metabolic phenotyping [181].

Dietary strategies emphasizing lower saturated fat intake together with greater consumption of plant-based foods support weight management while helping maintain nutritional adequacy [182,183]. Supplementation may provide additional benefit in selected situations, although evidence varies considerably between compounds. Vitamin D supplementation appears most useful in individuals with confirmed deficiency [184]. Myo-inositol has shown improvements in insulin sensitivity, particularly in women with polycystic ovary syndrome [185,186]. In contrast, alpha-lipoic acid may reduce oxidative stress markers, but its effects on clinically meaningful IR outcomes remain inconsistent [187,188].

Long-term adherence to lifestyle interventions remains a major challenge and is influenced by behavioral, social, and environmental factors. This partly explains why responses to intervention vary substantially between individuals and why more personalized approaches are often necessary. In some settings, additional clinical support may be required. Metformin remains a widely used first-line pharmacological therapy for T2D and has a well-established safety profile [11]. Its possible role in cancer prevention remains uncertain and continues to be investigated.

An important limitation across the intervention studies discussed in this chapter is the lack of adequately powered trials using cancer incidence as a primary endpoint. Although improvements in IR markers are consistently observed, their translation into reduced cancer risk remains inferred largely from epidemiological associations rather than demonstrated directly in intervention studies [3,150].

These findings support integrated prevention strategies that address metabolic, behavioral, and environmental determinants simultaneously.

## 7. Pharmacological Strategies in Insulin Resistance

### 7.1. GLP-1 Receptor Agonists and Dual Incretin Therapies

Although incretin-based therapies have demonstrated substantial benefits for weight reduction, glycemic control, and cardiovascular outcomes, their long-term effects on cancer incidence and cancer-specific mortality remain incompletely understood. Most available evidence originates from cardiovascular outcome trials, diabetes studies, and observational analyses that were designed to evaluate cardiometabolic rather than oncological endpoints. Consequently, while these studies provide robust evidence for metabolic and cardiovascular benefits, they cannot reliably establish cancer-related effects. Interpretation of both beneficial and adverse associations therefore requires caution, and extrapolation from cardiometabolic outcomes to oncological outcomes should be avoided. Long-term studies specifically designed to evaluate cancer incidence and cancer-specific mortality are still lacking.

The growing use of GLP-1RAs, particularly semaglutide, together with the introduction of the dual glucose-dependent insulinotropic polypeptide (GIP)/GLP-1 receptor agonist tirzepatide, has significantly changed the pharmacological management of IR. These therapies were originally developed for T2D, but their use has expanded rapidly into obesity treatment and broader cardiometabolic risk reduction [189,190].

This shift is increasingly reflected in international clinical guidance. In December 2025, the World Health Organization [WHO] issued its first global guideline formally recognizing pharmacotherapy as part of comprehensive obesity management. The recommendation builds on the 2022 WHO Acceleration Plan, which aimed to reduce global obesity prevalence by 5% by 2030. Current guidance supports the use of GLP-1-based therapies—including semaglutide, liraglutide, and tirzepatide—in combination with structured lifestyle intervention involving dietary modification, physical activity, and behavioral support [25,26]. These treatments are recommended for individuals with BMI ≥ 30 kg/m^2^, or ≥27 kg/m^2^ in the presence of obesity-related comorbidities. Clinically meaningful metabolic improvements are often observed within the first few months of treatment.

The metabolic effects of GLP-1RAs are mediated through several overlapping mechanisms. These agents enhance glucose-dependent insulin secretion while suppressing glucagon release, improving postprandial glycemic control. Delayed gastric emptying further reduces postprandial glucose excursions. Appetite suppression mediated through central nervous system pathways contributes to lower caloric intake and sustained weight reduction. This is particularly relevant because loss of visceral adipose tissue directly affects several mechanisms linked to metabolic dysfunction, including adipokine imbalance, chronic low-grade inflammation, and ectopic fat accumulation [191,192,193].

Their potential relevance may therefore extend beyond glucose regulation alone. By reducing hyperinsulinemia and improving adipose tissue function, incretin-based therapies may also influence components of the broader metabolic environment associated with carcinogenesis. At present direct evidence linking these therapies to reduced cancer incidence remains limited, and most proposed oncological effects remain mechanistic or observational rather than proven in long-term interventional studies.

### 7.2. Dual Incretin Therapy: Tirzepatide and Enhanced Metabolic Effects

Tirzepatide represents a further development of incretin-based therapy by combining GLP-1 receptor agonism with activation of the glucose-dependent insulinotropic polypeptide receptor (GIPR). Compared with GLP-1 receptor agonists alone, this dual mechanism appears to produce greater effects on body weight and metabolic regulation.

Much of the current evidence comes from the SURPASS and SURMOUNT trial programs. In SURPASS-2, tirzepatide produced larger reductions in HbA1c, fasting insulin, and body weight than semaglutide. These findings need to be interpreted carefully. The comparator was open-label semaglutide 1 mg rather than the higher doses now commonly used in obesity treatment and contemporary T2D management. This likely influenced the magnitude of the observed differences, and direct comparisons using equipotent doses are still lacking [189,191].

The stronger metabolic effects of tirzepatide are thought to reflect complementary receptor signaling. GLP-1 activity mainly affects appetite regulation, gastric emptying, and pancreatic insulin secretion, whereas GIP signaling appears to have additional effects on adipose tissue metabolism and lipid handling. Experimental studies suggest that GIP receptor activation may improve adipose tissue insulin sensitivity and metabolic flexibility, although much of this evidence remains mechanistic or preclinical [194,195,196]. In practice, the combined effect of these pathways may be particularly important for reducing visceral adiposity and improving the broader metabolic environment associated with IR.

This was reflected in the SURMOUNT-1 trial, where tirzepatide 15 mg produced a mean body-weight reduction of 20.9% over 72 weeks [197]. The scale of weight loss approached that seen after some bariatric procedures and was accompanied by improvements in glycemic markers, fasting insulin-related measures, and lipid profiles.

Whether these metabolic effects translate into lower cancer risk is much less clear. Observational studies have suggested reduced incidence of several obesity-related cancers among GLP-1RA users compared with individuals treated with insulin or metformin. Wang et al. [198], for example, reported lower risks of endometrial, colorectal, esophageal, and liver cancers. These findings are difficult to interpret because of substantial confounding. Patients prescribed GLP-1RAs often differ from comparator groups in body weight, baseline metabolic health, healthcare access, and treatment patterns. Differences in follow-up duration further complicate interpretation, making causal conclusions unreliable.

Other studies have reported less favorable findings. A nationwide emulated trial from Denmark found a modest increase in overall cancer incidence among long-term GLP-1RA users compared with users of dipeptidyl peptidase-4 (DPP-4) inhibitors [HR 1.35; 95% CI 1.05–1.73 at 6–10 years] [199]. The authors suggested that this may partly reflect survival bias rather than a direct carcinogenic effect, since individuals living longer also have greater opportunity for cancer detection. Importantly, no difference was observed in the composite outcome of death or cancer, and residual confounding—particularly related to BMI and underlying metabolic status—remains difficult to exclude.

Interpretation of observational studies is further complicated by potential surveillance bias. Individuals receiving GLP-1 receptor agonists often undergo more frequent medical evaluation, imaging procedures, and laboratory testing than untreated individuals. Increased healthcare contact may lead to earlier detection of pre-existing malignancies, thereby influencing reported incidence rates independently of any true biological effect. Consequently, differences in cancer incidence observed across observational studies may partly reflect differences in diagnostic intensity rather than direct effects of treatment [200,201].

Preclinical studies provide additional but indirect insight. In experimental models, GLP-1 receptor activation has been associated with anti-proliferative and pro-apoptotic effects in selected tumor cell lines and may influence tumor–stroma interactions, including extracellular matrix remodeling and cancer-associated fibroblast activity [202,203,204]. These findings suggest that incretin signaling could potentially alter the tumor microenvironment in ways relevant to tumor progression or immune evasion [205]. Their clinical significance, however, remains uncertain.

Clinical trial data available so far do not indicate increased cancer risk with tirzepatide. A recent meta-analysis of randomized controlled trials found no excess incidence across several cancer types, including breast, colorectal, pancreatic, thyroid, and gastric cancers [206]. Follow-up periods ranged only from 26 to 72 weeks, which limits conclusions regarding long-term oncological safety.

Another important limitation is the relatively short duration of follow-up in most available studies. Carcinogenesis is typically a long-term process that may evolve over many years or decades, whereas most randomized trials of GLP-1 receptor agonists report follow-up periods of only several years. Consequently, currently available data may be insufficient to detect either protective or adverse effects that emerge only after prolonged exposure.

Tirzepatide appears to be one of the most effective currently available pharmacological approaches for obesity-related metabolic dysfunction and IR. Available evidence remains insufficient to determine whether these metabolic benefits translate into meaningful changes in long-term cancer risk.

### 7.3. Safety Signals and Methodological Limitations in Cancer Research

Interpretation of the current evidence linking incretin-based therapies with cancer risk remains complicated by several important methodological limitations.

One major issue is that the lower cancer incidence reported in some observational studies may largely reflect weight loss and overall metabolic improvement rather than direct anti-tumor effects mediated through incretin signaling itself. This distinction is clinically relevant because it remains uncertain whether similar findings would be observed in individuals with IR who achieve only modest reductions in body weight.

Another unresolved question involves thyroid safety. Some observational studies have reported increased incidence of thyroid C-cell neoplasms among long-term GLP-1RA users. The signal is biologically plausible because GLP-1 receptors are expressed in thyroid parafollicular cells. Fragility index analyses suggest that these findings are statistically unstable and may lose significance under alternative analytical models [207,208,209]. Current evidence therefore remains inconclusive rather than clearly supportive of a causal association. Thyroid safety continues to be an area of active investigation. Rodent studies demonstrated C-cell hyperplasia and medullary thyroid tumors following prolonged GLP-1 receptor stimulation, findings that contributed to regulatory warnings for this drug class. Corresponding evidence in humans remains limited, and large clinical studies have not consistently demonstrated an increased incidence of thyroid cancer. Nevertheless, available follow-up remains relatively short, and continued pharmacovigilance is warranted [208,210,211].

Follow-up duration presents an additional limitation. Although randomized controlled trials have not demonstrated increased risk of gastrointestinal or pancreatic malignancies, most available studies are relatively short and therefore poorly suited for evaluation of long-latency oncological outcomes [212,213].

Pancreatic safety deserves particular attention. Early post-marketing reports raised concerns regarding possible associations between incretin-based therapies, pancreatitis, and pancreatic cancer. Subsequent randomized trials and meta-analyses have not provided consistent evidence supporting a causal relationship. Interpretation remains challenging because diabetes itself is associated with increased pancreatic cancer risk, and reverse causality may influence observational findings. Consequently, the absence of a detectable signal in relatively short-term studies should not be interpreted as definitive evidence regarding long-term pancreatic safety [210,213,214].

More broadly, much of the literature examining cancer outcomes with GLP-1RAs is observational and therefore vulnerable to several forms of bias. Confounding by indication, healthy-user bias, and differences in healthcare utilization or cancer surveillance are particularly difficult to control for in these populations [200]. Consequently, current evidence supports metabolic efficacy and short-term safety, but it does not establish a direct cancer-preventive effect.

Economic considerations further complicate translation into wider clinical practice. Cost-effectiveness analyses suggest that semaglutide and tirzepatide are unlikely to be cost-effective at current prices unless substantial price reductions occur [215]. Despite strong metabolic efficacy, treatment cost therefore remains a major barrier to accessibility, particularly in the context of large-scale prevention strategies.

In clinical practice, GLP-1RAs and tirzepatide are best viewed as supportive therapies rather than substitutes for lifestyle intervention. Their use is generally most appropriate in individuals with inadequate response to lifestyle modification or in those with substantial cardiometabolic risk. Treatment decisions also require individualized assessment because gastrointestinal adverse effects are dose-dependent, hypoglycemia risk may increase when these agents are combined with insulin secretagogues, and the long-term significance of the thyroid safety signal remains unresolved [207,213].

A broader issue emerging from this literature is the gap between mechanistic plausibility and direct clinical evidence. Incretin-based therapies consistently improve metabolic parameters linked to carcinogenesis, including HOMA-IR, visceral adiposity, and systemic inflammation. Whether these improvements ultimately translate into meaningful reductions in cancer incidence remains uncertain. Evidence supporting the metabolic and cardiovascular benefits of GLP-1 receptor agonists is substantially stronger than evidence regarding their effects on cancer risk. Improvements in body weight, glycemic control, visceral adiposity, and cardiometabolic outcomes have been demonstrated in large randomized clinical trials, whereas proposed anticancer effects are derived primarily from mechanistic studies, preclinical models, and secondary analyses of clinical datasets. Therefore, any conclusions regarding cancer prevention should currently be considered hypothesis-generating rather than established clinical evidence.

At present, GLP-1RAs and tirzepatide are best understood as highly effective metabolic therapies with possible—but still unproven—oncological implications. Their use specifically for cancer prevention would therefore be premature outside the context of long-term randomized studies incorporating dedicated cancer endpoints.

### 7.4. Pharmacokinetic Sustainability, Sarcopenic Risk, and Nutritional Displacement

Although obesity is increasingly recognized as a complex systemic disease, studies integrating metabolic, inflammatory, genomic, and microbiome-related processes remain relatively limited. Multi-omics approaches have therefore attracted growing interest as a possible framework for understanding why some individuals respond well to standardized interventions whereas others require more individualized strategies. In principle, combining genomic, metabolic, inflammatory, and microbiome data—particularly in longitudinal settings—may help clarify how both pharmacological and lifestyle interventions alter the biological environment associated with obesity and IR [216,217].

At present human evidence remains limited. Data examining genomic stability after semaglutide treatment are still scarce, and much of the mechanistic discussion relies on preclinical models. Experimental studies suggest that GLP-1 receptor agonists may reduce oxidative stress, increase antioxidant capacity, and influence expression of genes involved in DNA repair pathways [218,219,220,221]. These observations are biologically plausible, although their relevance to long-term human outcomes remains uncertain.

A related question is whether metabolic improvement necessarily translates into full biological recovery. Evidence from calorie-restriction studies suggests that weight loss can improve markers of genomic stability in individuals with obesity [222,223,224,225]. Several experimental and clinical studies indicate that inflammatory activation and oxidative DNA damage may persist even after substantial weight reduction, particularly in individuals with longstanding metabolic disease or multiple comorbidities. This persistence has been described as “obesogenic inflammatory memory,” reflecting the possibility that chronic low-grade inflammation and metabolic dysregulation may not be fully reversible despite normalization of body weight [226].

These observations complicate the assumption that weight loss alone is sufficient to eliminate downstream cancer-related risk. Long-term metabolic recovery may depend not only on reduction in adiposity, but also on sustained modification of inflammatory and oxidative pathways. In this context, preclinical evidence suggests that GLP-1 receptor agonists may attenuate inflammatory signaling, lipid peroxidation, and oxidative stress, with some measurable changes occurring relatively early during treatment [217,219,220].

Several practical concerns remain insufficiently studied. Rapid pharmacologically induced weight loss may also involve reductions in lean body mass, raising concerns regarding sarcopenia, nutritional adequacy, and long-term metabolic resilience, particularly in older individuals or patients with pre-existing frailty. In addition, marked appetite suppression may alter overall dietary quality if reduced caloric intake is accompanied by insufficient protein or micronutrient consumption. These issues remain underrepresented in current long-term outcome studies.

Incretin-based therapies provide substantial metabolic benefits, but their broader biological effects remain incompletely understood. Questions regarding genomic stability, inflammatory persistence, body composition changes, and long-term cancer risk still require confirmation in integrated longitudinal human studies.

## 8. Conclusions

The evidence reviewed throughout this manuscript supports the view that IR extends beyond a narrowly defined metabolic abnormality. Chronic hyperinsulinemia, oxidative stress, adipokine imbalance, low-grade inflammation, epigenetic alterations, and microbiome-related changes together create a systemic environment that may favor carcinogenesis. The relationship is clearly complex and cannot be reduced to a single pathway or mechanism. Current evidence supports a consistent association between IR and increased risk of several malignancies, although the strength of causal evidence varies substantially across cancer types. While multiple biological pathways provide plausible mechanistic links between IR and carcinogenesis, direct causal evidence remains heterogeneous across cancer types and is strongest for only a limited number of malignancies. MR studies provide suggestive support for some associations, particularly those related to hyperinsulinemia, whereas others become attenuated after genetic adjustment, indicating that residual confounding and coexisting metabolic factors may contribute to the observed epidemiological relationships. IR is therefore best understood as a biologically relevant modifier of cancer risk whose contribution likely varies across tumor types, rather than as a universal causal determinant.

One recurring theme across the literature is the disconnect between BMI and metabolic health. Individuals with normal BMI may still exhibit visceral adiposity, chronic inflammation, hepatic steatosis, and other features of metabolic dysfunction. This has important implications for screening and risk assessment because reliance on BMI alone may underestimate clinically relevant metabolic risk. The evidence reviewed here supports a broader metabolic phenotype approach integrating anthropometric measures, biochemical markers, and, where appropriate, dynamic tools such as CGM. This may be particularly relevant in individuals with normal-weight metabolic obesity, a phenotype that remains underrecognized in routine clinical practice.

Another important observation is that the biological consequences of obesity and IR may persist even after apparent metabolic improvement. Emerging concepts such as obesogenic inflammatory memory suggest that normalization of body weight does not necessarily imply complete reversal of inflammatory or oxidative pathways linked to long-term disease risk. This may partly explain why the relationship between metabolic improvement and cancer risk reduction remains difficult to define in clinical studies.

Lifestyle modification remains the most consistently supported strategy for prevention and long-term metabolic management. Dietary patterns such as the Mediterranean diet, regular physical activity, adequate sleep aligned with circadian biology, and stress reduction act across multiple interconnected pathways rather than targeting a single downstream mechanism. In contrast, pharmacological therapies—including GLP-1 receptor agonists and dual incretin therapies—provide substantial metabolic benefit but appear to function mainly as adjunctive tools. Their effects are dependent on continued treatment, long-term oncological implications remain uncertain, and current evidence does not support attributing direct cancer-preventive effects to incretin signaling itself.

The review also highlights several important limitations in the current evidence base. Much of the literature remains observational, with persistent vulnerability to residual confounding, selection bias, and reverse causality. Definitions and measurement of IR vary substantially between studies, while most intervention trials remain too short to evaluate cancer outcomes directly. In addition, many mechanistic claims regarding incretin therapies, microbiome modulation, and epigenetic recovery continue to rely heavily on preclinical or inferential evidence.

Several priorities for future research emerge from these gaps. These include prospective validation of integrated biomarker panels across diverse populations, longer-term mechanistic studies examining whether improvements in IR translate into reduced cancer incidence, and trials capable of distinguishing weight-loss-mediated from receptor-mediated effects of incretin therapies. Greater attention is also needed toward implementation strategies applicable to low- and middle-income countries, where the burden of both metabolic disease and cancer continues to rise rapidly.

This review supports the view that the metabolic environment itself should be considered central to understanding cancer risk in IR. Pharmacological therapies can modify parts of this environment, sometimes substantially, but they do not fully replace the broader biological effects of sustained lifestyle change. The most effective long-term strategy is therefore unlikely to involve lifestyle intervention or pharmacotherapy alone, but rather an integrated approach in which pharmacological treatment supports—rather than substitutes for—durable behavioral and metabolic change.

## Figures and Tables

**Table 1:** Comparative overview of epidemiological, mechanistic, and MR evidence linking IR to major cancer types.

Cancer Type	Epidemiological Evidence	Primary Proposed Mechanisms	MR Evidence	Consistency Across Studies	Interpretative Considerations
Endometrial	Strong; prospective and meta-analytic evidence links IR, insulin, C-peptide and IGF-related markers with increased risk [47,48,49]	Hyperinsulinemia, IGF-1 signaling, reduced SHBG/relative hyperestrogenism, PI3K/Akt/mTOR activation, frequent *PTEN* alterations [47,48,49,50,51]	Direct MR evidence not extensively reviewed here	High	Effects may be partly mediated through obesity and hormone-related pathways
Hepatocellular carcinoma	Strong association with metabolic dysfunction, MASLD, obesity and metabolic syndrome [36,52]	Hepatic IR, MASLD, fibrosis/cirrhosis, inflammation, ectopic lipid accumulation, and possible dysbiosis-related inflammatory signaling [36,52,53,54,55]	Limited direct MR evidence for IR-specific HCC risk	Moderate–high	Difficult to separate IR from MASLD/cirrhosis as intermediates
Pancreatic	Moderate; interpretation complicated by dysglycemia near diagnosis [34]	Hyperinsulinemia, β-cell/islet dysfunction, inflammation, possible IGF-related signaling [34,35]	Suggestive but inconsistent [11,35]	Low–moderate	Reverse causality is a major concern
Colorectal	Moderate; associations reported with metabolic syndrome, diet and sedentary patterns [36,37]	Hyperinsulinemia/IGF signaling, inflammation, dysbiosis, adipokine imbalance [53,54,55,56]	Mixed; MR did not confirm a clear direct IR–CRC association in available analyses [33]	Moderate	Diet, obesity, microbiome and sex may modify risk
Breast	Moderate; stronger in postmenopausal/NWO phenotypes [57,58]	IGF-1/PI3K/Akt/mTOR signaling, adipokine imbalance, inflammation, epigenetic effects in TNBC models [10,57,58,59,60]	Limited and not sufficiently characterized in the present review	Low–moderate	Strongly modified by menopausal status, ER subtype, adiposity and BMI

Abbreviations: IR, insulin resistance; IGF-1, insulin-like growth factor 1; SHBG, sex hormone-binding globulin; PI3K/Akt/mTOR, phosphoinositide 3-kinase/protein kinase B/mechanistic target of rapamycin; *PTEN*, phosphatase and tensin homolog; MR, Mendelian randomization; MASLD, metabolic dysfunction-associated steatotic liver disease; HCC, hepatocellular carcinoma; CRC, colorectal cancer; NWO, normal-weight obesity; TNBC, triple-negative breast cancer; ER, estrogen receptor.

## Data Availability

No new data were created or analyzed in this study. Data sharing is not applicable to this article.

## Abbreviations

The following abbreviations are used in this manuscript:

AMPK	AMP-activated protein kinase
*APC*	adenomatous polyposis coli
*ASXL1*	additional sex combs-like 1
BCAAs	branched-chain amino acids
BMI	body mass index
CBT-I	cognitive behavioral therapy for insomnia
CGM	continuous glucose monitoring
CHIP	clonal hematopoiesis of indeterminate potential
CI	confidence interval
DASH	Dietary Approaches to Stop Hypertension
DPP-4	dipeptidyl peptidase-4
*DNMT3A*	DNA methyltransferase 3A
EPIC	European Prospective Investigation into Cancer and Nutrition
FGF-21	fibroblast growth factor 21
FFAR2/FFAR3	free fatty acid receptor 2/free fatty acid receptor 3
GDM	gestational diabetes mellitus
GIP	glucose-dependent insulinotropic polypeptide
GIPR	glucose-dependent insulinotropic polypeptide receptor
GLP-1	glucagon-like peptide-1
GLP-1RA	glucagon-like peptide-1 receptor agonist
GLUT-4	glucose transporter type 4
HbA1c	hemoglobin A1c
HOMA-IR	homeostatic model assessment of insulin resistance
HPA	hypothalamic–pituitary–adrenal
HR	hazard ratio
hs-CRP	high-sensitivity C-reactive protein
IF	intermittent fasting
IGF	insulin-like growth factor
IGF-1	insulin-like growth factor 1
IL-1	interleukin-1
IL-1β	interleukin-1 beta
IL-6	interleukin-6
InsR	insulin receptor
IR	insulin resistance
IRS-1	insulin receptor substrate-1
JAK/STAT3	Janus kinase/signal transducer and activator of transcription 3
*KRAS*	Kirsten rat sarcoma viral oncogene homolog
LPS	lipopolysaccharide
MAPK	mitogen-activated protein kinase
MASLD	metabolic dysfunction-associated steatotic liver disease
MeSH	Medical Subject Headings
METS-IR	metabolic score for insulin resistance
MR	Mendelian randomization
NWO	normal-weight obesity
PI3K/Akt/mTOR	phosphoinositide 3-kinase/protein kinase B/mechanistic target of rapamycin
p70S6K1	ribosomal protein S6 kinase beta-1
PRAS40	proline-rich Akt substrate of 40 kDa
PRS	polygenic risk score
*PTEN*	phosphatase and tensin homolog
RBP4	retinol-binding protein 4
ROS	reactive oxygen species
RR	relative risk
SCFAs	short-chain fatty acids
T2D	type 2 diabetes
*TET2*	tet methylcytosine dioxygenase 2
TG/HDL	triglyceride-to-high-density lipoprotein ratio
TLR4	toll-like receptor 4
TNF-α	tumor necrosis factor alpha
*TP53*	tumor protein p53
TRE	time-restricted eating
TyG	triglyceride–glucose
UK	United Kingdom
UPFs	ultra-processed foods
WHI	Women’s Health Initiative
WHO	World Health Organization
WHtR	waist-to-height ratio
